# Supplementary material for: Best Practices on Radiology Department Workflow: Tips from the Impact of the COVID-19 Lockdown on an Italian University Hospital
Source: Healthcare (Basel). 2022 Sep 14;10(9):1771. doi: 10.3390/healthcare10091771 (PMC9498676; doi:10.3390/healthcare10091771)
Supplement: Supplementary file 1 [file healthcare-10-01771-s001.zip › healthcare-1906284-supplementary.pdf]

Table S1. Number of diagnostic investigations performed during the ordinary activity period (11 March 2019–26 May 2019) and the pandemic emergency (11 March 2020–26 May 2020) in the radiology department of our institution

	11 March 2019–26 May 2019		11 March 2020–26 May 2020		Comparison		
**Examinations by method**	**Number of examinations**	**Proportion**	**Number of examinations**	**Proportion**	**Difference**	**Percentage**	***p* Value**
Bone X-rays	11,559	22.8%	6957	20%	−4602	−39.8%	<0.001
Chest X-rays	8960	17.7%	5225	15%	−3735	−41.7%	<0.001
Other X-rays	1206	2.4%	641	1.8%	−565	−46.8%	<0.001
DXA	3329	6.6%	1738	5%	−1591	−47.8%	<0.001
Ultrasound	5510	10.9%	3343	9.6%	−2167	−39.3%	<0.001
Head CT	4250	8.4%	2200	6.3%	−2050	−48.2%	<0.001
Body CT	8159	16.1%	7245	20.8%	−914	−11.2%	<0.001
Chest CT	4709	9.3%	3845	11%	−864	−18.3%	<0.001
HRCT	189	0.4%	1644	4.7%	+1455	+769.8%	<0.001
Head MRI	665	1.3%	570	1.6%	−95	−14.3%	<0.001
Body MRI	919	1.8%	684	2%	−235	−25.6%	>0.05
Angiography	1144	2.3%	720	2.1%	−424	−37.1%	>0.05
**Total**	50,599	-	34,812	-	−15,787	−31.2%	-

Data are sorted by the employed method, and it is reported the proportion of each category. The comparison between the two periods is expressed in absolute numbers and percentage change. The "*p* value" column lists *p* values obtained in the post hoc analysis and adjusted according to the Hommel procedure.

Table S2. Diagnostic investigations performed within the ordinary activity (11 March 2019–26 May 2019) and during the pandemic emergency (11 March 2020–26 May 2020) by the two units of the radiology department of our institution

	GRU				ERU			
**Examinations by method**	**11 March 2019–26 May 2019**	**11 March 2020–26 May 2020**	**Difference**	**Percentage**	**11 March 2019–26 May 2019**	**11 March 2020–26 May 2020**	**Difference**	**Percentage**
Bone X-rays	5152	3654	−1498	−29.1%	6407	3303	−3104	−48.4%
Chest X-rays	2790	1161	−1629	−58.4%	6170	4064	−2106	−34.1%
Other X-rays	47	25	−22	−46.8%	1159	616	−543	−46.9%
DXA	3329	1738	−1591	−47.8%	-	-	-	-
Ultrasound	2958	2434	−524	−17.7%	2552	909	−1643	−64.4%
Head CT	2584	1902	−682	−26.4%	1666	298	−1368	−82.1%
Body CT	7108	6676	−432	−6.1%	1051	569	−482	−45.9%
Chest CT	4611	3643	−968	−21%	98	202	+104	+106.1%
HRCT	177	97	−80	−45.2%	12	1547	+1535	+12,791.7%
Head MRI	665	570	−95	−14.3%	-	-	-	-
Body MRI	919	684	−235	−25.6%	-	-	-	-
Angiography	863	676	−187	−21.7%	281	44	−237	−84.3%
**Total**	31,203	23,260	−7943	−25.5%	19,396	11,552	−7844	−40.4%

Data are sorted by method. The comparison between the two-time intervals is expressed in absolute numbers and percentage change.

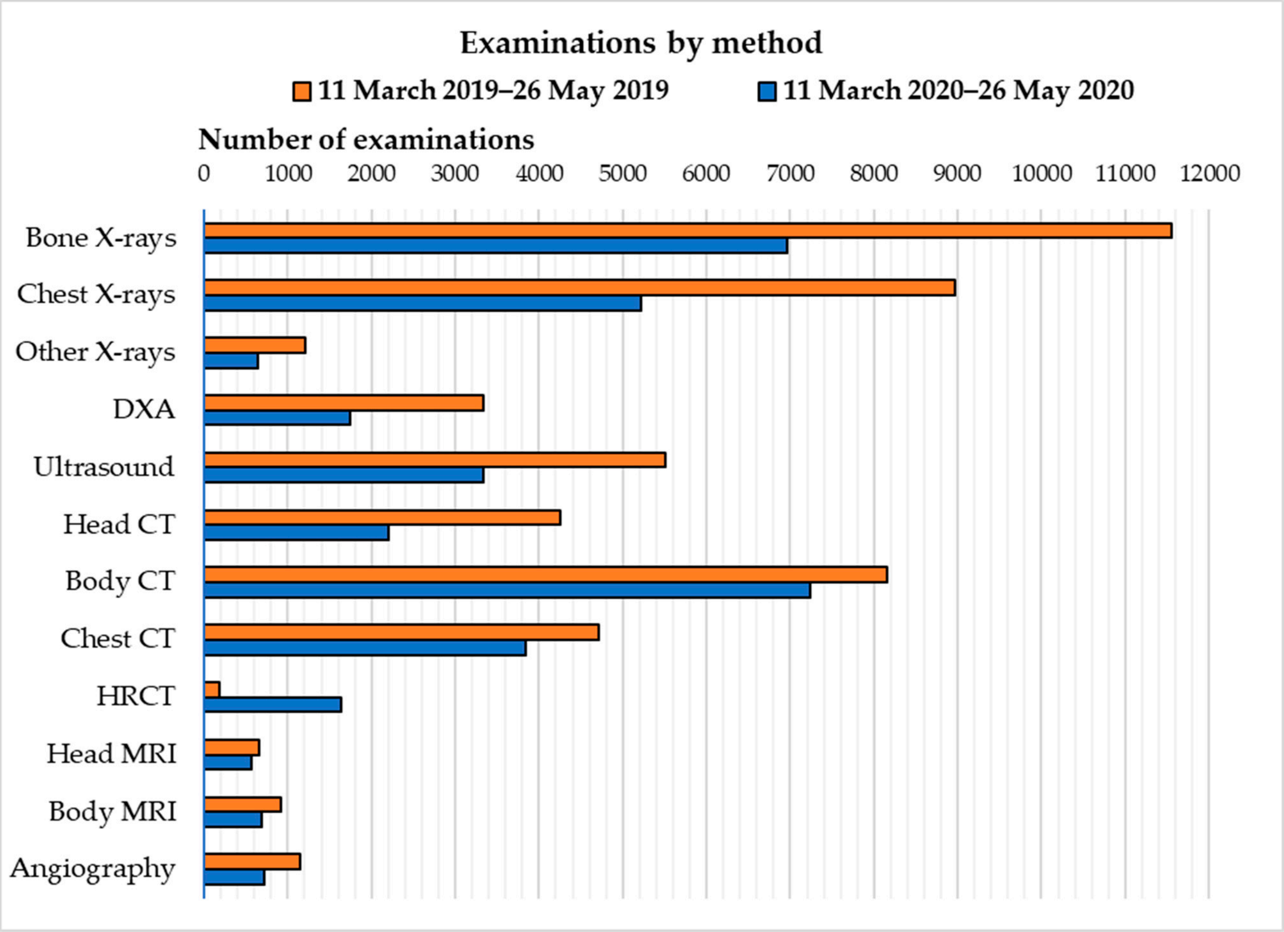

Figure S1. Graphical comparison between the number of diagnostic examinations, sorted by modality, performed during the ordinary activity period (11 March 2019–26 May 2019, represented in orange) and the pandemic emergency (11 March 2020–26 May 2020, represented in blue) in the radiology department of our institution.

Table S3. Weekly examinations performed to investigate traumas within the ordinary activity (11 March 2019–26 May 2019) and during the pandemic emergency (11 March 2020–26 May 2020) by the radiology department of our hospital.

	11 March 2019–26 May 2019	11 March 2020–26 May 2020	Comparison	
**Week number**	**Number of examinations**	**Number of examinations**	**Difference**	**Percentage**
Week 1 (11/03–17/03)	717	279	−438	−61.1%
Week 2 (18/03–24/03)	688	284	−404	−58.7%
Week 3 (25/03–31/03)	693	284	−409	−59%
Week 4 (01/04–07/04)	675	318	−357	−52.9%
Week 5 (08/04–14/04)	745	330	−415	−55.7%
Week 6 (15/04–21/04)	691	333	−358	−51.8%
Week 7 (22/04–28/04)	566	393	−173	−30.6%
Week 8 (29/04–05/05)	682	362	−320	−46.9%
Week 9 (06/05–12/05)	661	524	−137	−20.7%
Week 10 (13/05–19/05)	633	584	−49	−7.7%
Week 11 (20/05–26/05)	719	657	−62	−8.6%
**Total**	7470	4348	−3122	−41.8%

The comparison between the two periods is expressed in absolute numbers and percentage change.

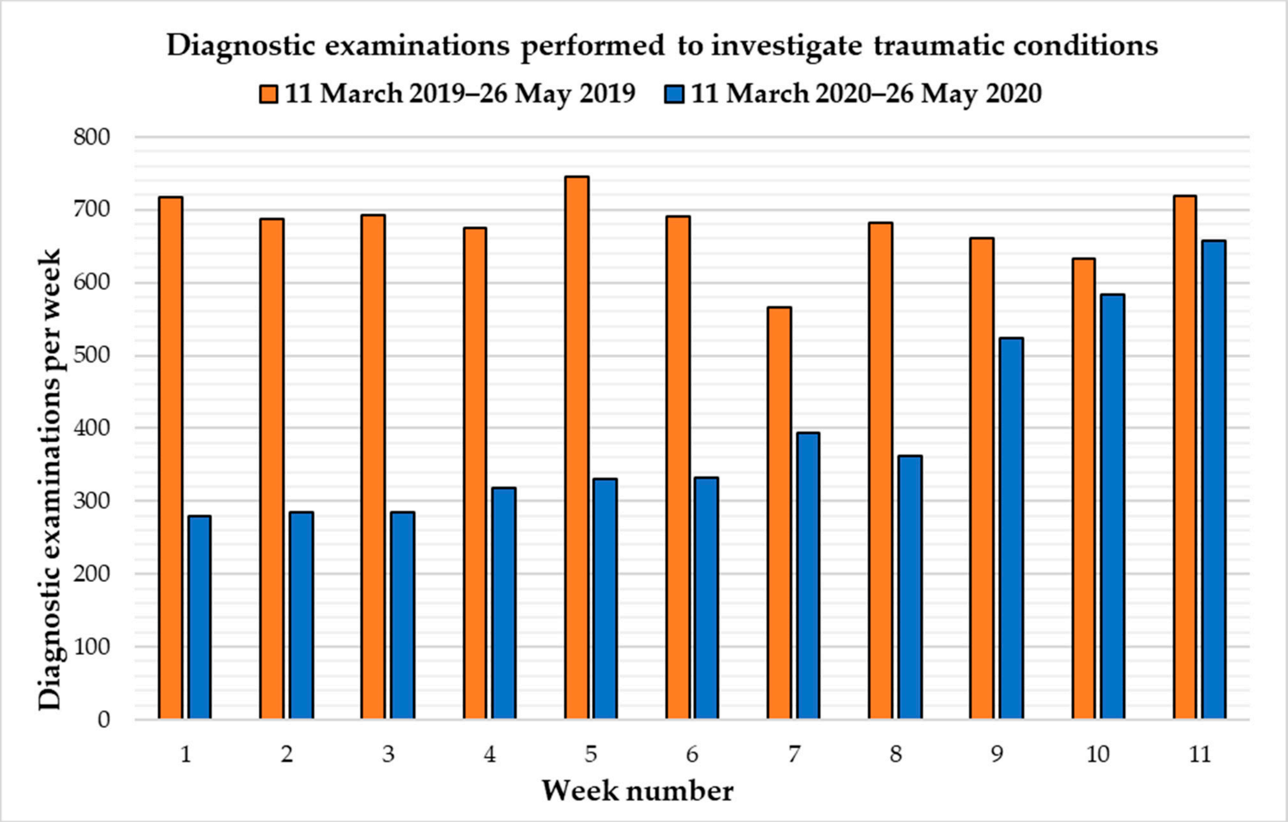

Figure S2. Graphical comparison between the number of weekly diagnostic examinations performed to investigate traumas during normal operativity (11 March 2019–26 May 2019, represented in orange) and the COVID−19 pandemic emergency period (11 March 2020–26 May 2020, represented in blue) in the radiology department of our institution.

Table S4. Number of diagnostic investigations performed during the ordinary activity period (11 March 2019–26 May 2019) and the pandemic emergency (11 March 2020–26 May 2020) in the radiology department of our hospital

	11 March 2019–26 May 2019		11 March 2020–26 May 2020		Comparison		
**Examinations by method**	**Number of examinations**	**Proportion**	**Number of examinations**	**Proportion**	**Difference**	**Percentage**	***p* Value**
**Bone X-rays**							
not trauma	6370	12.6%	4185	12%	−2185	−34.3%	>0.05
trauma	5189	10.3%	2772	8%	−2417	−46.6%	<0.001
**Chest X-rays**	8960	17.7%	5225	15%	−3735	−41.7%	<0.001
**Other X-rays**	1206	2.4%	641	1.8%	−565	−46.8%	<0.001
**DXA**	3329	6.6%	1738	5%	−1591	−47.8%	<0.001
**Ultrasound**							
not trauma	5149	10.2%	3181	9.1%	−1968	−38.2%	<0.001
trauma	361	0.7%	162	0.5%	−199	−55.1%	<0.001
**Head CT**							
not trauma	3549	7%	1726	5%	−1823	−51.4%	<0.001
trauma	701	1.4%	474	1.4%	−227	−32.4%	>0.05
**Body CT**							
not trauma	7805	15.4%	6932	19.9%	−873	−11.2%	<0.001
trauma	354	0.7%	313	0.9%	−41	−11.6%	0.008
**Chest CT**	4709	9.3%	3845	11%	−864	−18.3%	<0.001
**HRCT**	189	0.4%	1644	4.7%	+1455	+769.8%	<0.001
**Head MRI**							
not trauma	663	1.3%	567	1.6%	−96	−14.5%	0.001
trauma	2	0.004%	3	0.01%	+1	+50%	>0.05
**Body RMI**							
not trauma	915	1.8%	678	1.9%	−237	−25.9%	>0.05
trauma	4	0.01%	6	0.02%	+2	+50%	>0.05
**Angiography**	1144	2.3%	720	2.1%	−424	−37.1%	>0.05
**Total**	50,599		34,812		−15,787	−31.2%	

Data are sorted by the employed method, and it is reported the proportion of each category. Bone X-rays, ultrasound, head CT, body CT, head MRI and body MRI data are further split out based on the clinical question to differentiate examinations performed for traumas from those requested to investigate non-traumatic conditions. The comparison between the two periods is expressed in absolute numbers and percentage change. The "*p* value" column lists *p* values obtained in the post hoc analysis and adjusted according to the Hommel procedure.
